# How Wisdom Emerges from Intellectual Development: A Developmental/Historical Theory for Raising Mandelas

**DOI:** 10.3390/jintelligence9030047

**Published:** 2021-09-21

**Authors:** Andreas Demetriou, Antonis Liakos, Niyazi Kizilyürek

**Affiliations:** 1 Department of Psychology, University of Nicosia, Nicosia 1700, Cyprus; 2 Cyprus Academy of Science, Letters, and Arts, Nicosia 1700, Cyprus; 3 Faculty of History and Archaeology, National and Kapodistrian University of Athens, 15784 Athens, Greece; antoliakos@gmail.com; 4 Department of Turkish and Middle Eastern Studies, University of Cyprus, Kallipoleos 75, Nicosia 1678, Cyprus; niyazi@ucy.ac.cy

**Keywords:** intelligence, cognitive development, wisdom, education, conflict resolution, problem-solving, decision making, history-wars

## Abstract

This paper invokes cognitive developmental theory as a means for preparing citizens to deal with and resolve conflicts within or across nations. We take the centuries-old Greek–Turkish dispute as an example. We first outline a theory of intellectual development postulating that mental changes emerge in response to changing developmental priorities in successive life periods, namely, interaction control in infancy, attention control and representational awareness in preschool, inferential control and cognitive management in primary school, and advanced forms of reasoning and self-evaluation in adolescence. Based on this model, we outline a control theory of wisdom postulating that different aspects of wisdom emerge during development as different levels of control of relations with others: trust and care for others in infancy, taking the other’s perspective, reflectivity, and empathy in preschool, rationality and understanding the rules underlying individual and group interactions in primary school, and understanding the general principles of societal operation explaining the differences in approach and interest between groups in adolescence and early adulthood. We also outline the educational implications of this theory for the education of citizens by capitalizing on intellectual strengths at successive developmental periods to comprehensively understand the world and to act prudently when dealing with interpersonal and social or national conflict. Finally, the paper discusses the political constraints and implications of this theory. This is the first attempt to derive wisdom from the development of cognitive and personality processes from infancy through early adulthood and to connect it to serious world problems.

The modern world is a world of nation states and the foundation history of the nation states is a history of warfare. There is almost not a single nation state that did not emerge from war. Wars create a background of nationalism and uneven development which protract conflict and tension between or within countries for a long time after they end. Conflicts and tensions compromise life and well-being. One such conflict is the conflict between Greeks and Turks. The Greek nation state emerged from an independence war against the Ottoman Empire (1821–1828); modern Turkey was founded as a modern nation state after a war with Greece (1919–1921). Cyprus is a battlefield where the Greek–Turkish conflict is endlessly protracted. The national desire of Greek Cypriots to unite with Greece and counter the national desire of Turkish Cypriots for division of Cyprus between Greek Cypriots and Turkish Cypriots turned Cyprus into a hotspot of ethnic conflict, where the end of history (Fukuyama 1992) is still far away.

The Republic of Cyprus emerged as an independent state from a war against British colonialism (1955–1959) and was established as a constitutional bi-communal state governed by its two dominant communities, Greeks, about 80% of the population, and Turks, about 18%. Obviously, historical forces were too strong for the small young state to overcome. Even more so, the division between the two communities was institutionalized in the constitution: although political bodies, such as the government, the parliament, and public administration, were proportionally shared by the two communities, education was separate and under the jurisdiction of an independent body in each community. This was enough to perpetuate separatist and conflictual national narratives, undermining the very functioning of political institutions. The state collapsed soon after independence, in 1963, because of strong disputes about distribution of power between the two communities and lack of unifying pressures coming from the people. Since then, inter-communal tensions and conflict never ended and peaked in a coup and civil war within the Greek Cypriot community in 1974; as a result, a Turkish invasion followed, causing displacement of about one-third of the population and occupation of one-third of the island by Turkish forces since then. Negotiations about a political settlement of the dispute have continued under the auspices of the United Nations since 1963. In the words of a UN envoy, negotiations in Cyprus go well as long as they go on!

Our position here is that the consequences of this dispute for the life of people are disproportionately large. The issues at stake changed drastically over historical time, so that division and conflict cause more harm than profit for any other than those perpetuating their power by capitalizing on division, such as political parties and politicians across the divide. In fact, none of the causes that ignited the Greek revolution against the Ottomans or the wars in the late 19th and early 20th century between Greece and Turkey is currently active. However, the leadership and the peoples in Greece, Turkey, and Cyprus refrain from making decisions that would bring the conflict to an end. Legalistic disputes about distribution of power and jurisdiction in a common federal state draw on the centuries-old Greek–Turkish conflict which fuels current political disputes and conflicts of interests with historical arguments within and across the two nations. It is well known that perpetuating negative narratives about the other in groups with conflictual relations facilitates further conflict, preventing search for means that would facilitate overcoming the conflict (Psaltis et al. 2020). This background causes lack of trust and tolerance, ethnic and nationalist tension, and self-perpetuating hostility drawing on misdeeds against each other.

Some scholars proposed that the average level of intelligence in a population is related to the level of democracy, technological advancement, and prosperity and the ability to efficiently resolve tensions and disputes with other countries or between groups within a country (Rindermann 2018). The higher the level, the better it is for peace and well-being. Rindermann suggested that wars in the modern world abound in regions where secular intelligence is considerably lower than the standard average. However, it is questionable whether current theory of intelligence would suffice to help nations to overcome disputes. Rindermann’s argument above may be cyclical: there may well be social and political factors other than individual intelligence causing national or social success, which subsequently result in provisions raising individual intelligence, such as generalized education. In fact, international history abounds with examples where advanced nations made fatally self-defeating decisions, including Germany, Rindermann’s country.

We argue that no theory of intelligence or intellectual development suffices to generate solutions for serious social or political problems. To be useful for this sake, any theory would have to be much broader, accounting for individual and social development on several fronts, such as the following:

1. This theory would have to account for both (a) individual mental development and (b) the contribution of individual development to the functioning of the social groups and institutions at various levels, increasingly distancing from an individual’s own life, such as family, city, nation, and humanity. This requires understanding how cognitive, social, moral, and personality development interact. Currently, no comprehensive theory exists that would do justice to these interactions. Piaget’s theory might have been the closest approximation to it, delving into the common core of intellectual (Piaget 1968), social (Piaget 1951), and moral development (Piaget 1932). However, Piaget’s theory, gearing every aspect of understanding on the development of logical reasoning, is not accepted anymore. Current theories are too fragmented to satisfy the requirements above (see Demetriou and Spanoudis 2018).
2. This theory must have explicit provisions for education. Education needs developmentally informed guidance to (a) set clear goals, enabling smooth and functional integration of cognitive, social, emotional, and personality characteristics of individuals to serve social, political, and productive institutions and (b) efficiently implement them (Demetriou and Spanoudis 2018). Developing citizens must learn early in their life to consider conflicting points of view and preferences and integrate them in constructive syntheses, given the present situation but also the future.
  Finally, provisions are needed for the organization and functioning of social and political institutions, such as parliament and other decision-making bodies, to guide social and political goal setting and problem solving, enabling the handling of strong historical forces that compromise the present and future of people.

## 1. Historical and Epistemological Concerns: How Wisdom Emerges from Intellectual Development

The developmental model outlined here draws on two major sources: current theory and research on intellectual development and current theory and research on wisdom in dealing with important life problems and societal issues. We argue that intellectual development and the development of wisdom are more closely related than assumed in the literature. Our central position is that neither of these lines of research alone is sufficient for the attainment of the aims above. The theory of intellectual development focuses on cognitive processes in childhood and adolescence. The theory of wisdom focuses on a specific approach to problem-solving and decision-making in adulthood. Historically, the two fields operationalized constructs differently. However, thinking and problem-solving in actual life need not respect the boundaries of different research fields in psychological research.

The psychology of intelligence adopted Binet’s (Binet and Simon [1908] 1948).) priorities for studying intelligence in the early 20th century. These focused on mental processes which are important for school learning, such as reasoning, language, and comprehension of concepts. The field was admirably successful in identifying individual differences in these processes and directing educational decision-making accordingly (Anastasi 2005). The psychology of cognitive development, under the influence of Piaget (1968), focused on the development of reasoning (Flavell 1985). Notably, Piaget’s career as a researcher started in Binet’s laboratory. Additionally, as an epistemologist interested in the nature and origins of knowledge, Piaget prioritized the study of logical reasoning and understanding philosophically defined categories of reason, such as number, space, and causality, rather than life-important decision-making. Piaget’s ideas caused important progress in our understanding of cognitive development. However, both the individual differences and the developmental approach to cognition did ill-justice to problem-solving and decision-making of broad social or societal interest, the focus of this article.

The study of wisdom has a different history. In classical Greek philosophy, wisdom involves *nous* (mind), discerning reality, and *episteme*, knowing and reasoning on universal truths. Phronesis involves the ability to make correct decisions and reflect on experience for important life matters. Current psychological theory and research on wisdom includes both ancient constructs (Ferrari 2009). It is considered a complex state of mind and personality enabling adults to use personal life experiences and a broad knowledge basis to make prudent judgments about complex personal, interpersonal, and social problems, which are recognized as inclusive, balanced, moral, and beneficial for everyone involved (Ardelt et al. 2019; Baltes and Smith 1990; Staudinger 2008; Sternberg and Karami 2021).

Wisdom so conceived is intelligence at its best. The road to it is intellectual development, gradually constructing a scaffold of thought enabling one to discern and evaluate reality, know universal and historical truths and reason on them, make practically beneficial decisions given the situation, and reflect on them to become better for the future. Wisdom has been an object of research on adult development but not of development in childhood, *underestimating the fact that wise adults have been children*. The study of cognitive development focused on changes in cognitive processes from infancy through early adulthood, but it did not examine how problems of everyday childhood or adolescence are resolved. The position of this paper is that all aspects of wisdom have precursors in early cognitive development. Thus, our central concern is to ensure that wisdom will gradually emerge from each phase of intellectual development, preparing citizens to deal intelligently and prudently with personal, national, and international issues involving conflict.

## 2. Empirical Concerns: How Wisdom and Intelligence Are Related

All theories assume that wisdom includes intelligence and much more. This is reflected in the moderate correlation, circa.3, between various processes addressed by intelligence research, such as processing speed, reasoning, and vocabulary, and processes addressed by wisdom research, such as integration of multiple points of view, ethical considerations, and self-transcendence (Glück 2020a, 2020b; Grossmann et al. 2020). In neo-Piagetian theory, wisdom was associated with attaining formal or postformal reasoning (Kallio 1995). Therefore, there is wide agreement that “wisdom is not a form of intelligence, nor is intelligence a form of wisdom” Glück (2020a, p. 17). Wisdom requires the following processes: (1) cognitive processes underlying intelligence, such as reasoning, reflection, and metacognition; (2) creativity to conceive of new solutions and interpretations, if old solutions do not suffice; (3) knowledge of the persons and situations requiring solutions, “the pragmatics of life”; (4) products (ideas and solutions) balancing interests of the parties involved, which are recognized by all to advance common good; (5) the motivation and affectivity to act accordingly (Sternberg and Karami 2021; Sternberg et al. 2019). Processes in points 1 and 2 become increasingly integrated and abstract with development, accounting for individual differences in cognitive ability. Points 3 and 4 draw on the processes in 1 and 2 to acquire knowledge and skill in different conceptual or activity domains. Mastering these domains requires drawing on the first two types of processes in the fashion required for mastering other complex domains, such as science. Processes in point 5 relate to motivation and dispositions to get involved in all other processes.

Thus, intelligence–wisdom relations appear as an investment cascade: fluid reasoning together with certain interests and personality dispositions allow the construction of a broad knowledge base underlying crystallized intelligence, which may be extended, by some people, into the “big questions” of human existence. Some of these people may capitalize on their own and others’ life challenges and develop wisdom, if some intellectual possibilities are present and if they have certain personality qualities, such as openness, empathy, and self-reflectivity. According to Glück (2020a), wise people have high levels of intelligence, and they are open, reflective, empathic, and ethical; however, not all intelligent people are wise.

Some complex domains, such as science, are learned in systematic long studies. No such program exists for wisdom. Theories of wisdom assume that becoming wise requires long-term life planning, optimum life management, and life review, allowing to make meaning of past decisions and actions and optimize future ones (Baltes and Smith 1990; Staudinger 2008). Thus, learning by doing is assumed. The present article suggests that wisdom might develop more broadly if wisdom-building mechanisms are properly guided to become invested with the knowledge required together with handling the personality dispositions required to provide the emotional context and motivation for wise thinking and decisions. In other words, we suggest that education for wisdom must be part of a program aiming to support intellectual development.

The model of wisdom-based reasoning provides the operationalization of wisdom that is necessary for the development of a program for educating wisdom that draws on intellectual development. This model postulates that wise thinking draws on four aspects of cognition (Brienza et al. 2021; Grossmann 2017): (a) intellectual humility, emerging from recognition of the limits of one’s own knowledge; (b) recognition that there may be multiple points of view or perspectives or that a current issue of concern belongs to a broader context; (c) recognition that views or interests often change in social relations or in time; (d) systematic search for the integration of different opinions, which implies recognition of compromise as part of social decision-making. We show below that all four aspects of cognition required for wisdom-based reasoning are acquired in early and middle childhood as building blocks of cognitive development.

## 3. A Cognitive Developmental Theory for Intelligence and Wisdom

### 3.1. The Mental Architecture

The theory of intellectual development we employ makes four fundamental assumptions (Demetriou et al. 2018a; Demetriou and Spanoudis 2018).

First, the human mind involves mental processes that carry out different tasks for understanding and problem-solving, namely, control functions, enabling focusing on information and action critical at a given moment and updating as required; integration functions, enabling grasping relations, generalizing, and validly filling in missing information; and cognizance, enabling awareness of the objects of mental activity and mental processes.

Second, these processes are functionally intertwined, always operating together as a unit at various levels, from perception to abstract thinking; meaning-making emerges from their integrated functioning. This unit, named noetron, after nous, the Greek term for mind (Demetriou et al. 2021), operates as a “master-algorithm,” coordinating current goals and relational integration with awareness of mental processes, their objects, and contents. Noetron is constantly updated in reference to the results of ongoing activity, mental or actual, coordinating feedforward expectations with feedback from the results of activity; matching feedforward with feedback information enables subjective experiences ascribing intrinsic values to mental states and activities, allowing choices among them according to their value suggested by successes and failures. Any organism lacking this form of a unifying mental agency would not be capable of capitalizing on a balanced combination of past knowledge and experience for the sake of future-oriented understanding or action. Ideally, this combination optimizes choices by selecting the best fitting experience or concept to vary, based on how the future is conceived. This is a fundamental condition for the development of wisdom, which is an advanced aspect of comprehensive intelligent judgment.

Third, noetron expands with development, generating increasingly inclusive repertoires of action or thought choices. In cognitive science terms, this expansion gradually generates a Language of Thought including tokens of experience and action (representations), rules for their possible legitimate relations (reasoning) (Fodor 1975), and exemplars from experience providing ready-made frames for rule implementation. In other words, noetron is embedded into an expanding system integrating perceptual experiences into representations by various forms of rules underlying forms of reasoning, such as inductive, analogical, and deductive reasoning, and problem-solving scripts. Increasing flexibility in choosing and using different forms of reasoning and scripts implies increasingly efficient levels of control, for instance, going from action control in infancy to representational control in preschool to inferential control in primary school to truth control in adolescence.

Fourth, with age, the profile of mental ability varies depending on the developmental needs for exercising control at successive developmental phases. Developmental priorities change with the functional state of noetron. When a process is highly demanded for efficient noetron functioning, mastering this process becomes a dominant developmental priority. Changes in mastering this process become highly helpful for learning and highly predictive of learning outcomes in various domains, including school learning (Demetriou et al. 2019b, 2020a, 2020b, 2020c). After a critical integration point in satisfying functional demands, the two may vary independently, because the formation of mental ability shifts to other priorities, showing dependence on other processes (Demetriou et al. 2017; Demetriou and Spanoudis 2018). Developmental priorities and their relations with cognitive processes and wisdom are discussed below. Table 1 summarizes developmental priorities for cognitive and wisdom development and their educational implications.

### 3.2. Developing Mind: From Cognition to Wisdom

*Precursors of wisdom in infancy*. Episodic representations dominate in infancy. These are mental states preserving the spatial and time properties of actions and experiences. Thus, interaction control, allowing efficiency of actions on objects, is the developmental priority of infancy (Demetriou and Spanoudis 2018). Cognitively, mastering interaction control provides the background for understanding that one’s own actions may have implications for objects and persons and that taking control needs effort. However, strictly speaking, infants cannot be wise because they lack the representational resolution, integrative power, awareness, knowledge, and emotional stability required for wisdom. Mastering interaction control, the developmental priority of this cycle, sets the background for later attainment of wisdom. This background is set when interacting with objects and persons, exploring differences across them, engaging actively with them but staying calm when results violate expectations, seeking help to improve interactions, and enabling the infant to realize that some solutions are better than others and that attaining them requires trying out alternative solutions.

Individual differences in emotionality and affectivity appear early in infancy and these may interfere in mastering interaction control (Soto et al. 2011; Roberts et al. 2006). Differences between infants in reactivity to persons and objects suggest that some infants are more likely than others to enter the road to wisdom. Infants high in activity, attraction to novelty, and inclination to affiliate, but low in intense emotional reactions predisposing for self-control are more likely to enter this road. Infants low in these attributes and high in emotionality need special care to take control of their interactions in a context of emotional security. Emotionally, mastering interaction control lays the ground for trust and security in dealing with oneself and others. Failing to protect infants from these weaknesses may channel them away from coherent, balanced, and beneficial interactions with others later in life.

*Attaining intellectual humility, decentering, and recognition of uncertainty in early childhood*. Realistic mental representations emerge from episodic representation at 2–3 years and are associated with symbols, such as words and mental images. These dominate in preschool, from 2 to 7 years. Hence, representational awareness and attention control are the major developmental priorities in this period. Mastering attention control enables the attainment of more complex cognitive tasks, such as organizing action according to represented goals, following ongoing verbal interactions, and exploring the behavior and interactions between other persons and objects (Demetriou et al. 2018a; Diamond 2013; Zelazo 2015). Mastering symbol systems, such as language, and subjecting action under the control of representation, renders awareness of representations and control of attention important. Awareness of representations enables individuals to become social partners and negotiate each other’s views or intentions; it also provides a representational insight that views of reality are often mirrored in each person’s representations. Preschool children’s strong interest in the imaginary worlds of fairy tales and movies reflects humans’ emergent realization that the world may be represented by alternative, often surprising, ways (Hinchcliffe 2006) and that using them helps explore their possible differences and functionalities (Dubourg and Baumard 2021). Education of tolerance and empathy may capitalize on the preschooler’s discovery of imaginary worlds.

Notably, three of the four aspects of wisdom-based reasoning emerge in this period. The precursor of intellectual humility is children’s awareness of their ignorance. Children talk explicitly about their own and others’ knowledge and they admit their own ignorance (Harris et al. 2017). In addition, by the age of 4–5 years, children revise incorrect interpretations in the light of new information. By the age of 7–8 years, children are aware that visual or oral input can be ambiguous, and they differentiate the conditions of epistemic uncertainty from physical uncertainty (Robinson et al. 2006). This understanding predates recognition of uncertainty and change. Grasp of Theory of Mind (Wellman 2014) at 3–5 years strongly suggests that preschoolers understand that mental states and beliefs may differ between individuals, depending on the sources of information they have access to. These achievements are precursors of the recognition of others’ perspectives and relevance to context (Hughes and Leekham 2004). They also predate the reflective stance about oneself and others, a pivotal component of wisdom.

Children high in awareness of others’ mental and emotional states are more likely than children who are low in these processes to engage in activities leading to wisdom. These children may understand that activities and objects may be shared and that goals may be better attained by persons working together than one person working alone. If sociable, helpful, and generous to others, organized, systematic, planful, and creative, children may realize that they may have a role in leading shared activities and gaining satisfaction from success and praise. However, the reasoning needed to grasp the underlying causal relations between events and realities or between motives and their effects is still weak in this cycle. Moreover, control of social interaction and openness to experience are not yet well refined and consolidated (Demetriou et al. 2018a, 2018b; Roberts et al. 2006). Together, these weaknesses hinder the recognition of problems of importance to a group and designing broadly beneficial problem-solving activities. Thus, synthesizing beyond one’s own experience and perspective is limited.

For the present concerns, it would be useful if children in this cycle are guided to reflect on how their activities may be benevolent or may cause pain or distress in others (Weststrate et al. 2018). The keen representational interests of this cycle may be used to familiarize children with the experiences of children belonging to other groups and develop empathy for their agemates belonging to the other group. This requires ad hoc educational programs allowing children to listen to the stories *of the others from the others*, to remember the stories of others, and to hold group-specific narratives against each other. The aim is to build a conception of the world where self-centered attitudes and ethnocentric heroism are relativized vis-à-vis an overall human narrative where human life and general well-being dominate as standards for individual action (Kizilyurek 2019).

*Search for integration and compromise in primary school*. With representational awareness and attention control established by 5–6 years, priorities change in primary school, from 7 to 11 years. The relations between representations need to be worked out and accurately represented. Hence, cognitive priorities are redirected from knowing the represented world and coupling representations with the environment to the relations between representations and concepts themselves. Holding representations active for as long as required to process relations and connect them by inference are the major priorities of this period. Inductive inference is the major tool for grasping the relations between objects and concepts because it enables transfer of meaning from experience to novel situations. Therefore, processes for handling memory and inductive inference are the major contributors to the formation of general cognitive ability in this period. Explicit deductive reasoning emerges at the end of this period, from 8–11 years, reflecting the integration of inferential rules into a system where one representation may be systematically viewed from the point of view of others. It is notable that from 7–9 years, children recognize that two viewers or listeners might make different interpretations, depending on what information they have access to and the inference used to connect them (Kazi et al. 2019; Spanoudis et al. 2015).

Therefore, the fourth requirement for wisdom-based reasoning, the search for integration and compromise, emerges in this period of life. In rule-based thought, children have the mental capacity to employ reasoning to inter-relate activities and emotions with the requests or needs of others, such as parents, siblings, and school mates, when they diverge from intentions and wishes. This enables children to formulate concepts organizing one’s own experiences and action plans, understand the role of rules and prescriptions in one’s life, and use them to generate or negotiate solutions to problems. Rule-based thinkers may be aware of the underlying connections between concepts, events, and experiences, or actions, thoughts, and motives. They may also use previous knowledge or experience to anticipate consequences of actions or events. They can also be aware of different perspectives on the same event because they understand that information or knowledge causes differences in perceptions and attitudes, even if reasoning is the same. Therefore, rule-based thinkers may analyze problems rationally and can take a reflective stance toward others and problems, which is conducive to tolerance of alternative views and their possible synthesis into solutions going beyond one’s own preferences.

However, rule-based thought often fails to grasp higher-order relations highlighting links between seemingly unrelated rules or systems; in addition, rule-based thought lacks truth control tools protecting from fallacious reasoning. This often causes personal biases and perspectives to dominate, especially if promoted by authority, in individuals who are not socially oriented, emotionally stable, or open to novelty. For instance, lack of openness may hinder individuals from seeing problems from the perspective of others. These individuals may not avail themselves to opportunities conducive to wisdom. In short, many adults do not reach wisdom if they stay in rule-based thought.

To deal with the weaknesses of rule-based thought, children in this cycle must be induced to explicitly grasp the rules under which conflicting groups operate. They must reflect on how conflicting rules may cause conflicting actions, which may in turn cause pain and compromise the interests and well-being of all persons involved. They must also be induced to redefine each other’s rules so that commonly accepted and beneficial ones may be conceived and implemented. There is evidence that practicing distanced self-reflection in the third person about conflict situations increases wise judgments by widening one’s own often narrow self-focus (Grossmann et al. 2020). In addition, teaching by example and historical role models may be the method of choice to enable rule-based thinkers to see how important figures approached complex problems and experience, from their point of view, the benefits obtained for themselves (becoming important figures in society) and others (improvement of their condition) (see Grossmann 2017).

*Building wisdom-based knowledge in adolescence*. Intellectual changes are consolidated in adolescence when controlled reasoning is fully established. Overarching principles integrating rules into systems according to truth and validity dominate in adolescence. Principles enable thinkers to grasp when it is and when it is not possible to use an inferential rule to infer a state of reality based on this rule. Thus, a critical approach to reality is possible. These processes strengthen until middle age. In addition, cognitive self-evaluation and self-representation become powerful factors in the formation of cognitive ability among university-educated persons in middle age (Demetriou and Bakracevic 2009; Demetriou et al. 2017). In adulthood, persons must take control of their life, regardless of how far they have gone on each of the cognitive and personality development dimensions discussed above. Entering the worlds of work, family, and citizenship without the protective shields provided by parents and schools imposes strong cognitive, personality, and social requirements.

Therefore, principle-based thought is, by definition, an important tool for transcending one’s own subjectivity to view problems from the point of view of other persons or alternative contexts, a condition for wisdom (Ardelt et al. 2017, 2019; Demetriou n.d.; Kallio 1995). Sound deductive reasoning is a truth control system enabling persons to evaluate solutions and perspectives according to their truth, value, and scope in concern with the persons and stakes involved. The epistemic stance enables persons to realize that even the best solutions may be relative and subject to revision. Thus, at the individual level, these types of thought may guide the development of long-term life plans, such as choosing a course of studies or a profession, balancing value judgments for one’s own weaknesses and strengths vis-à-vis a preferred lifestyle or social role. At the social level, they may enable individuals to grasp assumptions and prescriptions of multiple contexts in which they live and enable problem-solving, generating solutions, decisions, and courses of action that are optimal for the individual and other persons or institutions affected.

Scholars argue that higher levels of mental functioning, such as principle-based thought, openness, and higher levels of ego development, are complementary aspects of the same construct: the mature mind (Costa and McCrae 1993; McCrae and Costa 1997) which creatively integrates cognitive, personality, and emotional trends and proclivities in dealing with problems (Demetriou et al. 2018c). It is notable that wisdom in later life is associated with openness to novelty, mindedness, and well-being coming from having purpose in life, satisfaction, positive relations with others, environmental mastery, and a general concern for the well-being of others (Wink and Staudinger 2016). In this cycle, adolescents must be induced to consider the multiplicity of factors that may cause a conflict, such as historical, religious, political, and economic reasons and grasp their underlying principles. They must also be induced to consider historical events and decisions of political or historical figures from the point of view of all of the actors involved. This would enable them to understand that wise decisions are often those which may not appear right at the time they are taken. They must also be induced to understand that the modern narratives about these events may serve purposes other than the interests and well-being of the individuals or institutions involved.

## 4. A Developmental Model for Wise Conflict Management in Schools

In conclusion, we have suggested that wisdom is an emergent system of control integrating cognitive, emotional, and personality abilities and attributes for the sake of efficient, constructive, and self-enhancing activities and relations with others. This is a long process starting from infancy, building on attaining control of processes dominating in successive periods of life. The central idea is that childhood is important for integrating wise judgment into spontaneous cognitive functioning because satisfying the developmental priorities of each cycle causes the necessary build-up of the cognitive and personality characteristics required for wise judgment. Mastering interactions with persons and objects (infancy) provides the background for knowing that one’s actions have implications for objects and other persons. Mastering executive control and becoming aware of mental worlds (early childhood) are necessary for judgments acceptable by many. Mastering inference and using it to organize understanding and action (middle and late childhood) is necessary for understanding that views and decisions build up mentally and are built on rule systems that may differ between persons or groups. Mastering principles, imagining possible worlds, and understanding that inference and interpretation may not always be true or valid (adolescence) is necessary for adopting the critical stance, enabling to analyze and explore truths and search systematically for truth. Mastering the art of balanced and constructive choices embedding judgments and decisions in societal, cultural, and historical perspectives requires knowledge and experience drawn from autonomous life (early adulthood). All of these must be acquired in a positive context enabling persons to take responsibility for their life, develop trust for others, and become motivated to be constructive for themselves and others.

The theory above may guide enhancement of tolerance for social and political differences and capitalizing on them for efficient and productive functioning in a world of differences and diversities in such a way that it may enhance learning in more classic school domains. Specifically, there is research showing that school performance at successive educational levels is best predicted by the processes associated with the developmental priorities of each developmental cycle: command of attention control processes and representational awareness at preschool, management of working memory and inductive reasoning in primary school, and mastery of deductive reasoning, language, and accurate self-evaluation at secondary school (Demetriou et al. 2019a, 2019b, 2020a, 2020b, 2021). Importantly, training of mental processes transfers to general cognitive ability only if aligned with developmental priorities: training on attention control and theory of mind in preschool (Rueda et al. 2012), training on working memory in primary school (Holmes and Gathercole 2014), and training on relational integration (Klauer and Phye 2008; Papageorgiou et al. 2016) and deductive reasoning schemes in secondary school (Christoforides et al. 2016).

## 5. Ending History Wars: Overcoming a Side-Effect of Democracy

National educational goals and priorities are shaped by the orientations and priorities of a society and the institutions implementing them (Demetriou 2013; Nisbett 2003). In modern states, educational goals and priorities exist at several levels, often in conflict with each other. Education is addressed to everyone, aiming to enable the understanding and use of complex knowledge and technology in societies where differences must be accepted and honored. However benign these aims are, they are often understood differently, and they are not unconditionally accepted by different groups and stakeholders. Parties and organizations compete for priorities and orientations of society, including education, because it prepares citizens for the future, thereby affecting their own existence and role. Modern states are governed by several authorities with time-limited overlapping mandates and mutually balanced powers. Therefore, shaping the aims of education and educational practices is complicated and tricky business.

It is assumed that the level and quality of education depends on the quality of democracy and vice versa. It is also assumed that the level and quality of education and democracy in a country causes improvement in the relations of this country with other countries, facilitating intelligent and wise analysis and resolution of disputes. Inversely, it is assumed that wars stem more frequently from authoritarian regimes than democracies. However, unfortunately, democracy may have its share in starting a war. In the national rivalry between Greece and Turkey, strangely enough, things proved more peaceful under authoritarian regimes or conservatist governments (Metaxas dictatorship 1936–1941, post-war Greek authoritarian governments 1945–1963 and 1967–1973) than under democratic rule and populist governments (George Papandreou’s government 1963–1967 and Andreas Papandreou’s governments in the eighties). This may be ascribed to the major influence that well-preserved nationalist ideas have on the public sphere and public opinion. These are preserved in the national narrative by education. They are often part of identity-building policies that shape the orientations of education in both Greece (Liakos 2008, 2011) and Turkey and Cyprus (Kizilyurek and Kizilyurek 2004). Individual identities and related attitudes and feelings are then exploited by politicians to increase their political appeal and access to power. In a sense, fossilized national narratives function as political traps channeling nations to wrong directions, because people’s rule and democracy are not always compatible with the relativization of national differences and the culture of rights of minorities and different groups within a nation or multinational entities, such as the European Union or multinational countries. National rivalry comes not from the elites, but from the peoples themselves.

History wars often reflect this situation and become a tool of democratic functioning, protracting real wars or social polarization within societies. Obviously, this interpretation does not imply that we favor authoritarian over democratic governance. On the contrary, it highlights the hurdles of democracy with the aim to remove them from political practices for the sake of its further development. It is the task of education to raise critical and wise citizens from infancy through adulthood. Hopefully, this would enable nations to properly understand and weigh social and international problems when dealing with conflict and rivalries. Even this benign aim may be disputed because it might be interpreted by some to endanger the continuation of a nation as a distinct entity in time and space. Thus, provisions are needed to ensure that wisdom-based education and upbringing are accepted and implemented by all nations involved. Perhaps, the European Union is the most interesting historical and political experiment designed to achieve these aims.

However, even this is disputed, especially in nations where national, social, and political orientations are not settled. In these nations, the ideal for a European citizen is often interpreted with caution, because no commonly accepted answer exists on how much a European identity may be integrated with national identity. In Greece, there is still insecurity toward Europe because many believe that it endangers national religion, values, and traditions (Stavridi-Patrikiou 2007). In Cyprus, there is an ongoing discussion that the establishment and success of the state of Cyprus will eventually compromise Greek identity, among the Greek Cypriots, or Turkish identity, among the Turkish Cypriots, in favor of a Cypriot identity. Political parties or other institutions, such as the Church, object to the development of new curricula in several subjects, especially history, language, and religion, because they are concerned that this is ill-intentioned, aiming to increase distance between Cyprus and Greece (or Turkey, depending upon the community). A few years ago, a new curriculum, developed under the leadership of the first author as the then Minister of Education and Culture, with the aim to develop a culture of historical reconciliation and mutual tolerance between the Greek and the Turkish Cypriot ignited fierce history wars among the Greek Cypriots and was strongly opposed by many, with the Church in the lead. The Archbishop of Cyprus went as far as to state in public that he “will invite people to burn the new history books” because they supposedly endanger national history, as he himself understands it. In fact, a new history war started again while this revision, about the teaching of the role of Ataturk during the Greek–Turkish war in the early 20th century, was in progress. Many stars would have to align before the implementation of psychological models, such as the present one, would change society. Without this alignment, these models may appear as an interesting academic exercise, at best.

Mandela was a wise man who led his country to end apartheid, a long and very painful conflict between the white and black populations of South Africa. Four Mandelas may be needed to resolve the Greek–Turkish dispute: one in Athens, one in Ankara, and two in Nicosia (a Greek Cypriot and a Turkish Cypriot). We do not have them yet. Perhaps, we will not have them for as long as populist nationalist elites across the divide instrumentalize historical and political disputes about nation and national identity. After all, Mandela emerged from South Africa’s prisons, not its schools! However, it may be time for countries that shaped history for centuries, such as Greece and Turkey, to protect their citizens and future generations from war, letting their Mandelas to emerge from their schools rather than from battlefields or prisons.

These Mandelas would have to understand the history of both nations in the long term; they must understand the reasons which caused their conflict in the past and the reasons which caused changes in their relations over the centuries. They must also understand that relations between nations change with changes in the wider historical and cultural context. For instance, neither Greece nor Turkey or Cyprus operate in the context of the Byzantine or the Ottoman empire; they rather operate in a completely different context, including the European Union. The distribution of power and influence is not primarily dependent on military power but on other forms of soft power, such as science and cultural productions. In this regard, cooperation is a win-win multiplicative factor of power and influence; military competition is a loss-loss factor weakening all nations involved in many different respects. If education would raise a majority of Mandelas in both nations (in all nations for that matter), then naturally Mandelas would emerge in the leadership of both nations, leading them to a new chapter in their history.

This paper outlined a theory of individual-social development that may help raise them and call them to service. Raising Mantelas in this fashion will enable nations to overcome historical conflicts in which they are trapped. Times scales in the resolution of these problems are much larger than individual lives. Thus, raising many Mandelas in education may be the optimal management of the future by societies trapped in their past.

## 6. Conclusions

In short, a theory of intellectual development that may lead to a deeper and more comprehensive understanding of social and political problems and to collective wisdom was outlined. To our knowledge, this is the first theory attempting to derive wisdom from the development of cognitive and personality processes from infancy through early adulthood and connect it to serious world problems. This theory aims to (i) advance a deeper understanding of social and political problems since early childhood, (ii) advance individual wisdom for the sake of social well-being and long-term human interests, and (iii) guide education to capitalize on developmental priorities to develop knowledge and mental skills conducive to a wise decision-making ability when dealing with conflicts and disputes. Obviously, this model needs to be tested empirically. Ideally, longitudinal evidence would show that individuals performing high in childhood on tasks addressed to recognition of one’s own ignorance, understanding others’ perspectives, and integrating rules in overarching systems serving the interests of different individuals are more likely to demonstrate wisdom in real-world problems in adulthood. This type of research is time and resource demanding. Alternatively, training of these processes would have to generalize to the four aspects of wise reasoning and cause changes in inter-group attitudes in the fashion found by Brienza et al. (2021).

It was also argued that long-held national narratives and convictions may be incompatible with attaining these aims. The mechanisms for choosing individual leaders in modern democracies and their political functioning when chosen may be incompatible with international and supra-social goal setting and dealing with conflicts. Leaders are mostly elected by nations or different political and social groups to maintain and enhance political, social, and economic interests of their constituencies. Therefore, the success of the present model would be facilitated if political institutions are founded that would ensure integrative, wise, and future-oriented policies and practices rather than policies trapped into the past and motivated to sustain historical divisions and tensions. Probably, countries need a Wisdom Authority to watch and guide analysis and decision-making of problems. Only Mandelas would have to stuff a country’s Wisdom Authority.

## Figures and Tables

**Table 1:** Dominant cognitive, wisdom, and educational priorities as a function of age.

Age	Cognitive Processes	Wisdom Processes	Education
Infancy (0–2 years)	Episodic representations; interaction control; attachment, reactivity, and emotionality precursors of personality.	Development of trust for others; feelings of mastery of interactions with persons and objects.	Controlled engagement in interactions with other persons and objects; systematic variation of action outcomes and management of ensuing emotionality.
Preschool (2–6 years)	Realistic representations; attention control and representational awareness; mastering symbol systems and imaginary worlds.	Recognition of uncertainly and limits of one’s own knowledge. Recognition of others’ perspectives. Emergent personality dispositions.	Guidance to reflect on one’s own activities and perspective; listen to the others’ stories; know the others’ heroes and well-being. Explore the others’ worlds.
Primary school (6–12 years)	Representation of rules; inference; inferential awareness; management of the inferential process. Personality characteristics tend to become established.	Specify the rules underlying different belief systems; search for integration and compromise; stay open and control emotionality.	Associate lines of argument with relevant belief systems; practice reflection from the other’s point of view. Elaborate on the differences between each other’s heroes and historical figures.
Secondary school (12–18 years)	Abstract representations; truth and validity control; accurate self-evaluation and self-concepts; personality dispositions are well established.	Transcend one’s own subjectivity; recognize relativity of positions and preferences; recognize differences between principles; specify the limits of different principles.	Examine cognitive, emotional, and societal implications for different options; explore differences between belief systems, e.g., national history, religion, social narratives, etc.; enhance interests and satisfaction of all.

## Data Availability

Not applicable.
